# Optimisation in general radiography

**DOI:** 10.2349/biij.3.2.e18

**Published:** 2007-04-01

**Authors:** CJ Martin

**Affiliations:** Health Physics, Gartnavel Royal Hospital, Glasgow, Scotland

**Keywords:** Radiography, dental radiography, X-ray film, automatic exposure control, anti-scatter grid

## Abstract

Radiography using film has been an established method for imaging the internal organs of the body for over 100 years. Surveys carried out during the 1980s identified a wide range in patient doses showing that there was scope for dosage reduction in many hospitals. This paper discusses factors that need to be considered in optimising the performance of radiographic equipment. The most important factor is choice of the screen/film combination, and the preparation of automatic exposure control devices to suit its characteristics. Tube potential determines the photon energies in the X-ray beam, with the selection involving a compromise between image contrast and the dose to the patient. Allied to this is the choice of anti-scatter grid, as a high grid ratio effectively removes the larger component of scatter when using higher tube potentials. However, a high grid ratio attenuates the X-ray beam more heavily. Decisions about grids and use of low attenuation components are particularly important for paediatric radiography, which uses lower energy X-ray beams. Another factor which can reduce patient dose is the use of copper filtration to remove more low-energy X-rays. Regular surveys of patient dose and comparisons with diagnostic reference levels that provide a guide representing good practice enable units for which doses are higher to be identified. Causes can then be investigated and changes implemented to address any shortfalls. Application of these methods has led to a gradual reduction in doses in many countries.

## INTRODUCTION

Radiography using film has been the primary tool in radiology for over a century. The radiation dose to the patient was given only minor consideration during the early days. As the number of examinations performed has increased and data on the long term risks of cancer arising from ionising radiation exposure has emerged, more attention has been focussed on keeping the doses received to a minimum.. National programmes were set up to assess doses from radiological examinations in developed countries. A survey carried out in the UK in the early 1980s showed that mean doses from similar radiographic examinations varied by a factor of seven between different hospitals [1] and a factor of a hundred was present between doses for individual patients. The National Evaluation of X-ray Trends (NEXT) program has painted a similar picture in the United States [2]. It was apparent that in many hospitals the dose levels were much higher than required to provide a sufficiently high-quality image for the radiologist to make a diagnosis. Since that time more emphasis has been placed on the need to optimise imaging conditions to minimise the risk to patients from radiation exposure [3].

The quality of an image and the anatomical detail seen within it depend on the properties of the imaging system and the radiation used. In general, use of more radiation will improve the quality of the image within certain limits, but will give the patient a higher radiation dose, although other factors also need to be considered. The important aspects of optimisation are to first recognise the level of radiographic image quality that is required to make a diagnosis. Next to determine the technique that provides that level of image quality with the minimum dose to the patient. The image quality should be sufficient to ensure that any clinical diagnostic information that could be obtained is imaged. However, the radiation dose to the patient should not be significantly higher than necessary. Finally the procedures should be reviewed from time to time to ensure that any dose reduction that has been achieved does not jeopardise the clinical diagnosis.

## ASSESSMENT OF RADIATION DOSE AND IMAGE QUALITY

Before discussing optimisation in radiography in more depth, it is worth considering briefly the ways in which dose and image quality can be measured. There are several different quantities that are used for evaluating doses to patients. The dose quantities that can be measured for radiographic exposures are the entrance surface dose (ESD) and the dose-area product (DAP). The ESD is the dose to the skin at the point where an X-ray beam enters the body and includes both the incident air kerma and radiation backscattered from the tissue. It can be measured with small dosimeters placed on the skin, or calculated from radiographic exposure factors coupled with measurements of X-ray tube output [4, 5, 6]. The DAP is the product of the dose in air (air kerma) within the X-ray beam and the beam area, and is therefore a measure of all the radiation that enters a patient. It can be measured using an ionisation chamber fitted to the X-ray tube. DAP and ESD can be used to monitor, audit and compare radiation doses from a wide variety of radiological examinations. To provide a comparator that could be used to achieve more uniformity in patient doses for similar examinations in different hospitals, diagnostic reference levels (DRLs) or guidance levels for particular examinations have been established in terms of the ESD or DAP. National DRLs have been set up or proposed by various organisations based on surveys of doses in a large number of hospitals [7, 8, 9]. Conventionally, the third quartile of the distribution of mean doses from each of the hospitals in the survey for the particular examination is used as a guide in setting the DRLs, so that mean doses for three quarters of the hospitals are below the DRL and one quarter of them are above [7, 10]. DRLs proposed for a selection of radiographic examinations are given in Table 1 for adults and Table 2 for children [11, 12]. The mean dose in a hospital for a selection of patients of average weight should be less than the relevant DRL. If the DRL is exceeded, this should trigger an investigation into whether further optimisation is needed. This paper reviews the various factors that could contribute to higher doses, which may need to be considered if the dose for an examination is found to be too high. A requirement for countries in the European Union to establish and use DRLs has been included in a European Directive [13]. Adoption of an optimisation strategy with national and local DRLs in the UK has lowered patient doses, as demonstrated by the gradual reduction in third quartile values derived from UK-wide surveys of mean doses for large numbers of hospitals by the National Radiological Protection Board (NRPB) (Table 3) [9, 11].

**Table 1:** Suggested values for diagnostic reference levels for radiographs of adult patients [11 and local data]

**Radiograph**	**ESD per radiograph(mGy)**	**DAP per radiograph(Gy cm2)**
Skull AP/PA	3	0.7 *
Skull LAT	1.5	0.5 *
Chest PA	0.2	0.12
Chest LAT	0.7	0.5 *
Thoracic spine AP	3.5	1.5 *
Thoracic spine LAT	10	2.0 *
Lumbar spine AP	6	1.6
Lumbar spine LAT	14	3
Lumbar spine LSJ	26	3
Abdomen AP	6	3
Pelvis AP	4	3

* Based on patient data collected in West of Scotland

**Table 2:** Suggested DRLs for individual radiographs on paediatric patients in terms of ESD [8, 12]

**Radiograph**	**1 y**	**5 y**	**10 y**	**15 y**
Skull AP/PA	0.8	1.1	1.1	1.1
Skull LAT	0.5	0.8	0.8	0.8
Chest AP/PA	0.05	0.07	0.12	
Abdomen AP/PA	0.4	0.5	0.8	1.2
Pelvis AP	0.5	0.6	0.7	2.0

**Table 3:** Third quartile values for ESDs (mGy) from NRPB reviews of UK national patient dose data [10]

**Radiograph**	**Mid-1980s survey**	**1995 review**	**2000 review**
Skull AP/PA	5	4	3
Skull LAT	3	2	1.6
Chest PA	0.3	0.2	0.2
Chest LAT	1.5	0.7	1
Thoracic spine AP	7	5	3.5
Thoracic spine LAT	20	16	10
Lumbar spine AP	10	7	6
Lumbar spine LAT	30	20	14
Lumbar spine LSJ	40	35	26
Abdomen AP	10	7	6
Pelvis AP	10	5	4

Effective dose attempts to provide a quantity, related to the risk of health detriment for a reference patient in terms of stochastic effects in the long term [14]. It equates the uniform dose to the whole body that would have a similar level of risk and takes account of doses to radio-sensitive organs in different parts of the body. However, the effective dose can only be derived from calculations. These are based on computer simulations of the interactions of X-rays as they pass through the various organs within the body. The organ doses must be estimated from measurable dose quantities resulting in large uncertainties in the values. Effective dose is useful for comparing doses from different types of examination in general terms for a reference patient, and assessing changes in the dose for a reference patient during the process of optimisation. Another quantity that is simple to derive and can be equally useful, but whose application has declined since the introduction of effective dose, is the energy imparted to the body by an X-ray exposure [15, 16]. This includes all the energy absorbed from an X-ray beam, and so gives a more complete picture of the relative harm than a measurement of ESD, but does not include the complications and approximations involved in the calculation of effective dose.

A radiographic image provides a representation of the spatial distribution of tissue components as variations in the optical density of film. Image quality can be quantified in terms of the characteristics; contrast, sharpness (or resolution), and noise. Contrast is a result of the different attenuations of X-radiation in tissue; sharpness is the capability to display small details; and noise refers to the random fluctuations across the image that tend to obscure the detail. Evaluation and diagnosis from the image requires structures of interest to be distinguished against the background. The difference between the film optical density of a structure of interest and that of the background can be thought of as the signal. Random fluctuations across the film can occur, which are superimposed on the image. These are referred to as noise, and result from a number of causes; quantum mottle due to statistical variations because of the finite number of photons; the granularity or finite grain size of the film; and anatomic variations in structure density through the tissue. The fluctuations affect the detection of low contrast structures. An optimised radiograph should be limited by quantum mottle. If quantum mottle is less than noise due to one of the other factors, then it is likely that the film / screen combination chosen is slower than necessary and the dose to the patient is greater than it needs to be. Objective methods of evaluating image quality measure the imaging performance in terms of the signal reproduction for details of different sizes, using quantities such as the modulation transfer function (MTF), and their visibility within the noise generated by the imaging system, using the detective quantum efficiency (DQE) [17, 18]. The DQE characterises the performance of the radiographic system in terms of the efficiency with which the image information is reproduced. These variables are used in standards laboratories and film company research laboratories for evaluating the performance of screen / film systems.

Medical image quality is related to the subjective interpretation of visual data. It represents the clinical information contained in the image. It is more important that the observer interprets the image appropriately than whether the appearance of the image is pleasing to the eye. The ideal set of parameters to describe image quality should measure the effectiveness with which an image can be used for its intended purpose. However, since the interpretation and diagnosis made from an X-ray involve subjective opinions from the radiologist, results are likely to vary at different centres. Guidelines have been set up by the European Commission (EC) for assessing the basic aspects of quality for clinical radiographic images dependent on technique and imaging performance [8, 19]. This sets out diagnostic requirements against which the observer can judge an image. These requirements include aspects related to physical technique and production of anatomical structure for a normal individual. Visualisation of anatomical structures which should be clearly observed in particular types of radiograph are assessed as well as image detail which should be reproduced. This ensures that techniques employed within a department provide clinical images of acceptable quality and any changes made to reduce doses do not have a detrimental effect on the clinical image. Examples of criteria that may be affected by choice of technique are given in Table 4. The EC guidelines [8] also contain examples of parameters that are considered to represent good radiographic technique (Table 5)

**Table 4:** Image quality criteria relating to X-ray equipment and exposure factors

**Chest PA**
Image criteria Visually sharp reproduction of: • the vascular pattern in the whole lung, particularly the peripheral vessels • The trachea and proximal bronchi • The borders of the heart and aorta • The diaphragm and lateral costo-phrenic angles Visualisation of: • the retrocardiac lung and mediastinum • the spine through the heart shadow
Important image details Small round details in the whole lung, including the retrocardiac areas; • High contrast: 0.7 mm diameter • Low contrast 2 mm diameter Linear and reticular details out to the lung periphery: • High contrast: 0.3 mm in width • Low contrast 2 mm in width
**Lumbar spine AP/PA**
Image criteria Visually sharp reproduction of the pedicles Reproduction of: • the intervertebral joints • the spinous and transvers processes Visually sharp reproduction of the cortex and trabecular structures Reproduction of: • the adjacent soft tissues, particularly the psoas shadows • the sacro-iliac joints
Important image details: 0.3-0.5 mm
**Pelvis AP**
Image criteria Visually sharp reproduction of: • the sacrum and its intervertebral foramina • the pubic and ischial rami • the sacroiliac joints • the necks of the femora • the spongiosa and corticalis, and of the trochanters
Important image details: 0.5 mm
**Skull lateral**
Image criteria Visually sharp reproduction of: • the outer and inner lamina of the cranial vault, the floor of the sella, and the apex of the petrous temporal bone • the vascular channels, the vertex of the skull and the trabecular structure of the cranium
Important image details: 0.3-0.5 mm

Results from both practical measurements and theoretical simulations are included in this paper to illustrate how the different factors involved in radiographic imaging affect the radiation dose to the patient. Exposures have been made on anthropomorphic phantoms to mimic radiographs performed with a range of tube potentials for a selection of projections. ESDs have been assessed and effective doses have been calculated. Spreadsheet calculations have been performed using data sets for X-ray spectra, and these have been used to predict the responses of film / screen systems with different tube potentials, and various filter options [20].

## PHOTON FLUENCE OR RADIATION INTENSITY

### Screen / film combinations

**Figure 1:**
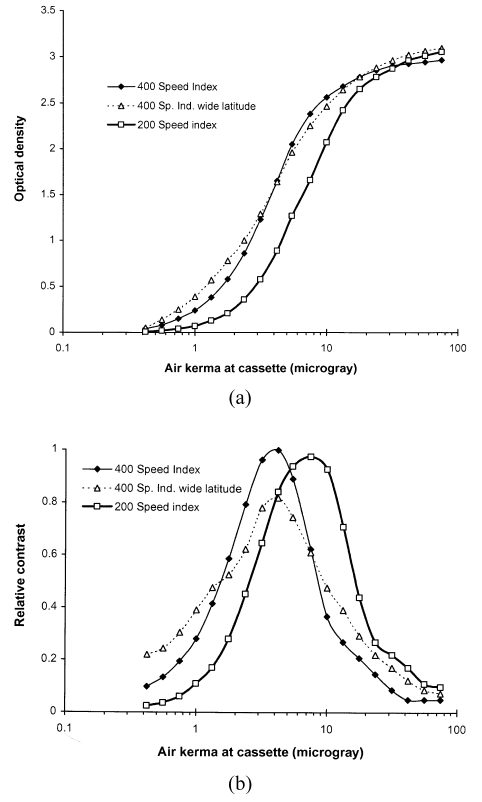
Curves showing the properties of three films, two 400 speed index screen / film combinations with differing contrasts and film latitudes and one 200 speed index; a) characteristic curve showing the variation in optical density with air kerma incident on the cassette, and b) variation in contrast with incident air kerma.

The most important factor in the optimisation of conventional radiography is the choice of screen / film combination. The X-ray film is sandwiched between two screens inside a light-tight cassette. Each screen has a layer of a fluorescent phosphor, such as calcium tungstate or gadolinium oxysulphide, which converts X-ray photons into visible light photons. The spectral emission of the phosphor must be matched to the sensitivity of the film. Calcium tungstate, the traditional phosphor used in radiographic screens, emits blue light, terbium activated gadolinium oxysulphide, the phosphor used in rare earth screens manufactured by Kodak, Agfa and Fuji emits green light, and yttrium tantalite used by Du Pont in the Ultravision system emits ultraviolet light. Using a film in the wrong type of cassette would require an X-ray exposure of higher magnitude. A definite relationship exists between film optical density and radiation exposure for every screen / film combination, and this can be described by a characteristic curve. Examples are given in Figure 1a. Exposures must be constrained to within the range that will produce perceptible differences in film blackening for the human visual system. Thus the range of exposures to be used for any radiograph is pre-determined through the choice of fluorescent screen and film. While the dynamic range of film is very limited, digital imaging systems have wide dynamic ranges, enabling images with acceptable contrast to be obtained for a broad range of exposure levels.

**Figure 2:**
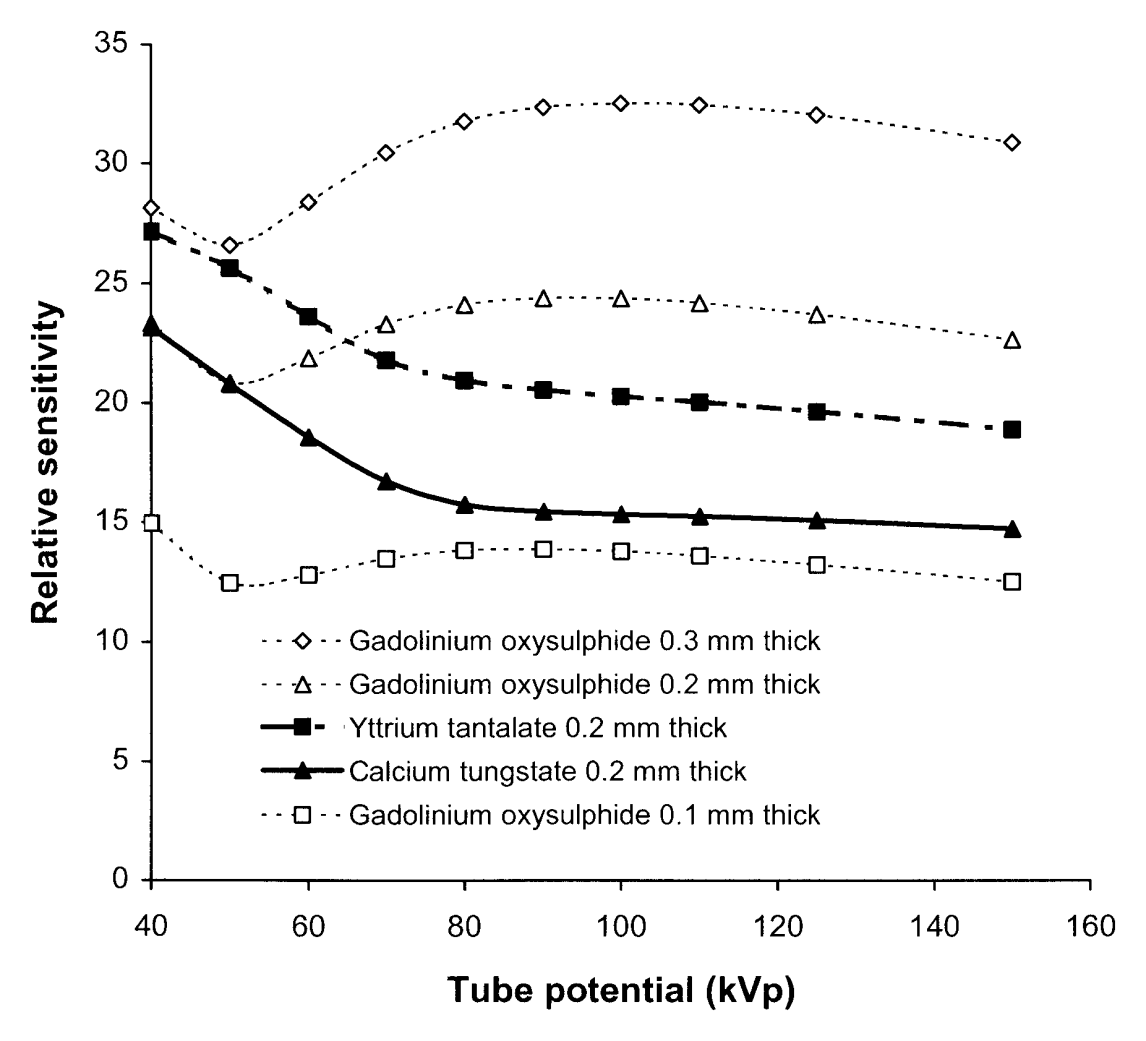
Relative sensitivity for different tube potential X-ray beams for three phosphors used in conventional radiography; gadolinium oxysulphide, yttrium tantalite and calcium tungstate, each 200 μm thick. Relative sensitivities are also shown for different thicknesses of gadolinium oxysulphide phosphor. The X-ray spectra applied are those transmitted through 2.5 mm aluminium and 200 mm of water.

The sensitivities of different systems depend on the absorption properties of the phosphors. Relative sensitivities for a selection of phosphors to X-ray beams corresponding to different tube potentials are shown in Figure 2. Gadolinium and other rare earth atoms have greater absorption at photon energies above 50 keV than calcium tungstate, and as a result, the rare earth screens have better sensitivities for X-ray beams with tube potentials above 70 kVp. Yttrium tantalate has an X-ray photon energy dependence that is similar to calcium tungstate, but a higher absorption and sensitivity. The thickness chosen for the phosphor layer is a compromise between radiation dose and image quality. A thick screen will have a high efficiency for conversion of X-rays to light, but the image will be more blurred as some of the X-ray photon interactions will occur further away from the film and therefore the light photons produced will spread out further before reaching the film. Thin screens result in better resolution but require a higher radiation exposure. Sensitivities of gadolinium oxysulphide screens of different thickness are compared in Figure 2. The sensitivity of screen / film combinations is quantified in terms of a speed index, which relates to the reciprocal of the dose to the cassette (in mGy) required to produce an optical density of 1.0 above the base plus fog level. It is analogous to the film speed employed in conventional photography. A higher speed index corresponds to a faster film and less radiation will be required to produce an image, although the radiograph will be noisier (more grainy). A speed index of 400 has been the standard for general radiography in Europe since the late 1980s [8, 21]. However, before that time, speed index combinations of 200 were widely used and may still be the combinations employed in many countries. In the UK, 200 speed index film cassettes will be used for imaging fine detail, for example to visualise fractures in the extremities. 600 or 800 speed indices are very high speed systems, but may be satisfactory for some applications such as lumbar spine and lumbar sacral joint imaging [22, 23].

**Figure 3:**
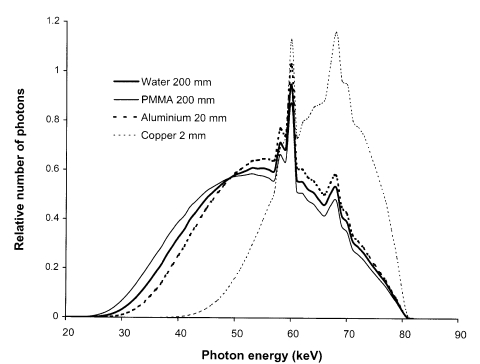
Spectra transmitted through filters comprising 200 mm water, 200 mm Perspex, 20 mm aluminium, and 2 mm copper, which are often used to harden X-ray beams to simulate the X-ray spectra transmitted through patients.

Knowledge of the speed index of a film/screen combination plays an important role in optimisation, and a combination used with a low speed index is the most probable reason for exposures being high. Speed indices may be measured by deriving characteristic curves from films exposed to a range of dose levels. Various phantoms may be used to simulate the spectrum transmitted through the body and methods have been described in the literature [24]. Although 20 cm thick water or Perspex provide the closest approximation to the spectrum, a 20 mm thick aluminium phantom may provide a more practical alternative with a transmitted spectrum not too dissimilar from that of tissue (Figure 3). The transmission of copper, which is sometimes used for such measurements does not resemble tissue transmission as closely and will therefore give slightly different results. Sections of the film to be tested should be exposed to a range of air kerma levels, covering the full range of optical density from 0.2 to over 2.0. This is normally achieved by using a single film, and covering parts of the cassette with lead. Higher exposure levels may be achieved by leaving parts of the film uncovered for several exposures. Measurements of optical density can then be plotted against the air kerma that is incident on the cassette in the form shown in Figure 1a. An assessment of the speed index can be calculated from the reciprocal of the air kerma in mGy to give an optical density of 1.0 above the film base plus fog level.

The image contrast and the range of exposure levels to be reproduced are also important factors in the choice of screen / film system. The contrast is defined in terms of the slope of the characteristic curve (Figure 1a), which can also be quantified from the measurements described. A high contrast screen / film combination equates to a steep slope for which the film optical density varies rapidly with dose and tissue attenuation (Figure 1b). The contrast is linked to the relative amount of film blackening produced by different exposures and also to the differences in tissue attenuation that can be imaged. A high contrast film will produce better visualisation of subtle variations in tissue structure. However, a high contrast film will be unsuitable for imaging the chest, which contains tissues such as the lung, heart and spine that have very different attenuations. For this, a combination that will give an acceptable level of contrast over a wide range of exposure, referred to as a wide latitude film, is required (Figure 1). For intra-oral dental radiography, film sensitive to X-rays and backed by lead foil is placed in the mouth. E-speed film is recommended and this should not require the ESD to be greater than 2.5 mGy.

### Exposure control

To produce an image on film with an acceptable level of contrast, the exposure must be within a relatively narrow range of doses. The exposure factors used will be optimised through the experience of the radiographers, and exposure charts employed for each X-ray unit. The charts provide a guide to the best factors for different examinations for a patient of standard build. However, adjustments will need to be made for patients of different sizes.

**Figure 4:**
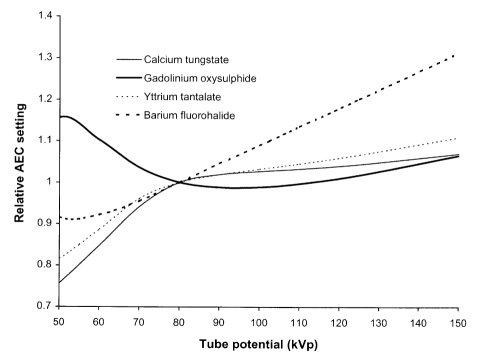
Relative dose setting for an AEC device against tube potential for different phosphors each 200 μm thick, for X-ray spectra transmitted through 2.5 mm aluminium and 200 mm of water. Data are also shown for a 200 μm thick barium fluorohalide phosphor used in computed radiography.

To achieve a consistent exposure level, an automatic exposure control (AEC) device is usually employed in fixed radiographic imaging facilities. This comprises a set of X-ray detectors behind the patient that measure the radiation incident on the cassette. The detectors are usually thin ionisation chambers. Exposures are terminated when a pre-determined dose level is reached, thereby ensuring that similar exposures are given to the image receptor for imaging patients of different sizes. The important parameter involved in radiographic image formation is optical density, so film is used in setting up the AEC to give a constant optical density. The variation in relative exposure with tube potential is determined by the phosphor sensitivities (Figure 2). Plotting this data relative to the response at 80 kVp gives an indication of the variation in relative dose level with tube potential that is required when setting up AECs for different screen / film combinations (Figure 4). A spectrum similar to that transmitted through tissue should be used to set up an AEC system. Two hundred millimetre thick phantoms of water or Perspex are suitable for this, but if these are not available, then 20 mm of aluminium provides an acceptable alternative, giving a similar transmitted spectrum (Figure 3). A metal filter will be thinner and can be attached to the X-ray tube light beam diaphragm and so may be more convenient to use. However, filters of heavier metals such as copper are less suitable. This is because the filter must be very thin (e.g. 0.5 mm copper), to obtain an energy spectrum similar to that transmitted through 200 mm of water and the exposure times required to give usable film densities will be too short for standard X-ray generators. If thicker filters are used (e.g. 2 mm copper), the spectra differ from those transmitted through tissue (Figure 3). Therefore the dependence of film density on kVp is different from that for tissue.

**Figure 5:**
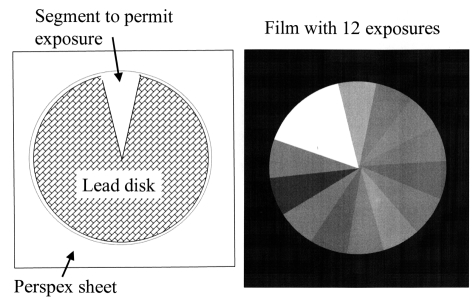
Tool that can be used to record a number of X-ray exposures on one film for setting up radiographic unit AEC devices, showing (Left) the tool itself and (Right) a simulation of the image that might be obtained.

Parts of the cassette used for the assessment can be shielded to avoid use of new films for each X-ray exposure. A 1.5 mm to 2 mm thick lead disc, about 150 mm in diameter, from which a 10^o^ – 20^o^ segment has been cut, provides a useful tool for this (Figure 5). A positioning device consisting of a Perspex sheet, with a hole to place the disc within and a lip to hold it in place can be fixed on the surface of the cassette. The disc should be rotated through the segment angle between exposures so that a new segment of film is left unshielded each time. Different kVs and different phantom thicknesses should be used to cover the range of exposures required in clinical practice, with different AEC chambers selected to terminate the exposure, to test the performance under a variety of conditions simulating clinical practice. A careful record of the disc orientation and sequence of exposures must be made to allow interpretation. Coins or other metal objects placed in suitable positions provide useful markers on the film. Most radiographic units have standard relationships between exposure and tube potential relating to different screen / film systems, and the purpose of the measurements is to ensure that the most appropriate AEC relationship for the combination is selected.

Radiography is performed using mobile equipment, but the quality of the image is likely to be lower, because radiographic cassettes cannot be aligned as accurately as with a fixed unit, and the distance of the cassette from the X-ray tube will be variable [25]. An AEC cannot be used to terminate the exposure, so an exposure chart is essential. The output of mobile X-ray units is lower than for fixed ones, so the range of exposures that can be obtained is limited and longer exposure times may be required. Therefore, mobile radiography should only be used in situations when an examination on a fixed installation is not feasible.

Exposure levels for most radiographic techniques will be determined by air kerma measurements in the X-ray beam. Evaluating a dose for a panoramic dental X-ray unit is more difficult, as it is necessary to integrate the dose from the exposure over the period during which the X-ray tube is moved around the head. This is measured in terms of the dose in the X-ray beam multiplied by the beam width or ‘dose-width product’. The dose-width product can be determined from the beam characteristics at the receiving slit measured over one rotation, either by a small detector that can be placed at the centre of the X-ray beam, multiplied by the beam width, measured using film, or using a CT chamber attached perpendicular to the slit [26]. A reasonable value for the dose-width product is 75 mGy mm, or it can be multiplied by the height of the X-ray beam at the receiving slit to derive the DAP, for which the DRL will be of the order of 100 mGy cm^2^.

## X-RAY BEAM QUALITY

Radiation quality refers to the proportions of photons with different energies within an X-ray beam. The contrast between different structures in an X-ray image results from removal of photons from the primary beam. The radiation quality influences the image quality and radiation dose through the mechanisms by which the X-ray photons of different energy interact with the tissue [20, 27]. Few photons with energies below 30 keV will be transmitted through 20 cm of tissue or water (Figure 3), so metal filters are placed in the X-ray beam which remove more of the low energy photons. X-ray beams which contain more photons with energies between 30 keV and 50 keV give better image contrast, but a greater proportion of the photons are absorbed in the body, so a larger radiation intensity must be used to obtain sufficient photons to form an image. The radiation quality of the X-ray beam chosen for each radiological examination should be selected to achieve the best compromise for the clinical task. The factors that determine the radiation quality are the tube potential and the beam filtration. Factors recommended by the EC for radiographs of a patient of standard size are given in Table 5

### Tube potential

**Figure 6:**
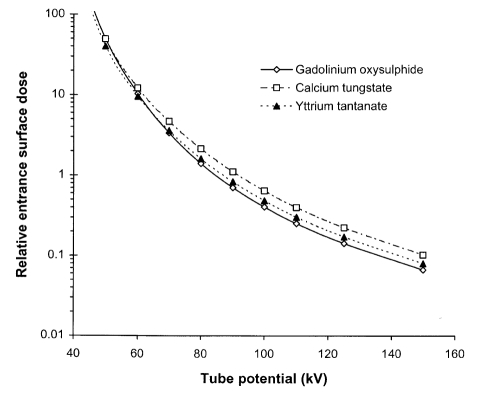
Graphs of relative entrance surface dose against tube potential to obtain an image for a 200 mm thickness of soft tissue with three phosphors used in radiographic cassettes; gadolinium oxysulphide, calcium tungstate and yttrium tantanate. All calculations are performed for 200 μm thick phosphor layers and an X-ray beam filtered by 2.5 mm of aluminium.

**Figure 7:**
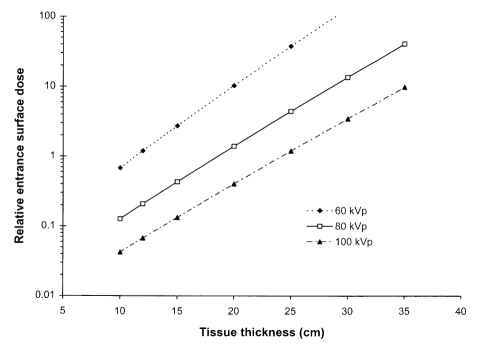
Graph of relative entrance surface dose against thickness of tissue to obtain an image with a rare earth screen / film combination using different tube potentials.

The potential applied to the X-ray tube determines both the maximum photon energy and the proportion of high energy photons. The optimum potential will depend on the part of the body being imaged, the size of the patient, the type of information required and the response of the image receptor. Figure 6 shows the reduction in incident air kerma that is the result of using higher tube potential to gain the same level of film blackening, for different phosphors used in radiographic screens. The ESD will be reduced by about 50% if the tube potential is increased by 10 kV. Figure 6 also shows that the exposure required for a calcium tungstate screen would be typically 50% higher than for a gadolinium oxysulphide screen. Tube potentials used for radiographic examinations have been established through experience. 80 kV to 85 kV are typical values used for radiographs of the abdomen, pelvis and lumbar spine antero-posterior (AP) views for an average patient. X-ray beams with tube potentials of 50 kV to 60 kV will give better contrast, but fewer photons will be transmitted. These are used for thinner regions of the body, such as the arms, hands and feet. 85 kV to 90 kV X-rays will provide better beam penetration and a lower radiation dose, but poorer contrast. They are employed for thicker, more attenuating parts of the body, such as the lumbar spine lateral projection. Standard kV ranges have been recommended for a selection of common radiographic examinations based on practices in different countries (Table 5) [8]. Patient doses will be significantly greater if lower tube potentials than those recommended are used [5, 28]. As the thickness of the part of the body to be imaged or of the patient increases, the exposure will need to be increased (Figure 7). If the tube potential remains the same, the ESD is about doubled for each additional 50 mm of tissue in the range 80 kVp to 100 kVp, and will increase by 2.5 to 3 times at 60 kVp. Therefore the tube potential will normally be increased for larger patients to keep the dose at a reasonable level. Using a higher tube potential results in poorer contrast and tends to produce more scatter, further reducing the image quality.

**Figure 8:**
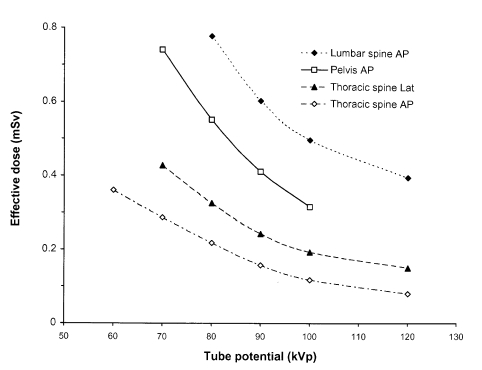
Variation in effective dose for a reference patient with tube potential for several different projections. The results were obtained through imaging of anthropomorphic phantoms with a rare earth screen / film combination, and calculation of values for the effective dose based on measurements of the entrance surface dose using conversion factors [29].

The reduction in effective dose when tube potential is increased is less than that in ESD or DAP, because the surface dose is proportionately higher with lower tube potentials. Plots showing the reduction in effective dose for a reference patient with tube potential were derived from practical experiments using anthropomorphic phantoms and are shown in Figure 8. The ESD was measured and then multiplied by conversion coefficients to estimate the effective dose [29]. Anthropomorphic phantoms are useful because they provide a normal anatomy reference patient which can be X-rayed multiple times using different exposure factors. Artificial lesions can be manufactured using Blu-Tack or similar materials to allow assessment of details of varying size. This type of investigation may be undertaken for assessment and evaluation of possible alternative techniques and therefore contribute to optimisation.

### Filtration

Thin sheets of metal such as aluminium or copper are incorporated into diagnostic X-ray tubes to reduce the proportion of low energy photons, as few are transmitted through the patient and contribute to the image. A filter equivalent to at least 2.5 mm of aluminium is incorporated as standard into medical X-ray tubes and is required by national guidance [30, 31]. Copper will absorb a higher proportion of the lower energy photons than aluminium, which contribute significantly to patient ESD. The disadvantage of using copper filters is that an increased tube output is required to compensate for the additional attenuation. With tube potentials of 70-80 kV, reductions of over 50% in ESD and 40% in effective dose can be achieved by using a 0.2 mm thick copper filter, but the tube output would need to be increased by about 50% to provide the necessary air kerma level [20]. Rare earth filters such as erbium have been investigated as possible alternatives to copper for imaging thinner tissue structures in paediatric and dental radiography. The advantage was their perceived ability to attenuate higher energy photons (>60 keV), and lower energy ones, therefore providing a narrower energy spectrum. However, apart from dental radiography, they have not provided significant advantages over copper filters.

## OTHER FACTORS IN OPTIMSATION

### Scattered radiation and use of low attenuation components

Once an X-ray beam has been transmitted through a patient, no new information can be obtained, but different components can be selectively emphasised or suppressed. Radiation scattered from tissues within the body, increases the level of random background noise on the film and this degrades the visibility of low contrast details. The amount of scattered radiation can be reduced by means of an anti-scatter grid. The grid consists of a plate containing thin strips of lead lying perpendicular to the plate surface, which are sandwiched between a low attenuation inter-space material such as fibre or paper. X-ray photons that do not change direction as they are transmitted through the patient pass between the lead strips with little attenuation, whereas scattered photons are more likely to be attenuated by the lead strips. The lead strips may be parallel, but can be angled towards the focal spot of the X-ray tube to improve transmission. The grid attenuates the transmitted primary beam and removes scattered radiation, which requires a higher intensity X-ray beam resulting in a higher radiation dose to the patient. The inter-space material may be carbon fibre, low attenuation plastic, or aluminium, and the grid cover is often made of aluminium [32]. Using a grid with aluminium interspaces is likely to double the dose required without a grid, whereas a grid with fibre inter-space only increases the dose by about 50%. However, the amount by which the radiation level will need to be increased depends on the grid characteristics and the tube potential. In paediatric radiography, carbon fibre or other low attenuation material should be used for all components between the patient and the film, because the attenuation of the low kVp X-rays is greater. For example, patient exposure can be increased by about 5% at low tube potentials by an aluminium grid cover. Use of a low attenuation X-ray couch is also particularly important for paediatric radiography, as some couches can attenuate the beam by 20% to 30%.

**Figure 9:**
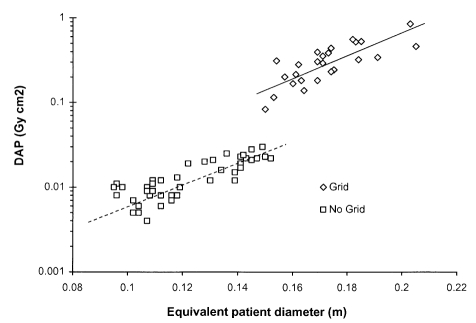
Plot of DAP against equivalent patient diameter for pelvis radiographs of children [33]. Smaller children were placed directly on the radiographic cassette on top of the X-ray couch, while for older children the cassette was placed in the bucky tray behind an anti-scatter grid.

The decisions to use a grid or not, and the choice of the technique employed for scatter reduction are important and involve image quality and dose, and depend on the application. An anti-scatter grid should only be used if more diagnostic information will be obtained as a result. A radiographic examination of an adult abdomen performed without a grid is unlikely to show the detailed tissue structure required for diagnosis, whereas a similar examination for a young child is likely to be satisfactory, because much less scattered radiation is generated. In Figure 9 data on the DAPs for paediatric examinations of the pelvis are plotted as a function of the equivalent diameter of the patient, calculated from the weight and height [33]. The figure displays discontinuity in the dose data that corresponds to the size of patient for which the technique was changed. A grid was not used for smaller children who were placed directly on the cassette on the X-ray couch. As a result there was no attenuation by the grid or X-ray couch and the focus to film distance was reduced. Older children were placed on the standard X-ray table and a grid was employed.

For examinations where a grid is used, the choice of grid characteristics is important for optimising imaging performance [32, 34]. The anti-scatter grid types are categorised by the strip density N, the grid ratio r, and the material used for the interspace. The strip density, i.e., the number of strips per cm, determines whether the grid can be used in a stationary mode or must be moved during the exposure to prevent the appearance of lines on the image. Strip densities over 60 strips per cm do not require mechanical movement. The grid ratio between the depth of the lead strips in the direction of the X-ray beam and the width of the fibre interspace perpendicular to the beam direction determines the effectiveness of the grid in removing scattered radiation, but also affects the transmission of the primary beam. Typical values for the grid ratio are between 18:1 and 8:1 with higher ratios being more effective in removing scatter. For thicker parts of the body, such as the lumbar spine, for which there are relatively large amounts of scatter, the use of high grid ratios (e.g. 16:1 – 18:1) with a high tube potential will give better image quality. When there is less scatter, a lower grid ratio (8:1) can be used, with a lower tube potential to give the desired contrast level. A 12:1 grid may provide an acceptable compromise allowing a range of general radiography with a single grid.

Much debate has taken place about the best technique for chest radiography over the years. Less scatter is produced by the lung that has a lower tissue density, but the heart and spine scatter radiation. A lower tube potential (65 kV – 70 kV) without a grid was the favoured technique in the UK, to give good detail in the lung, but a higher tube potential (110 kV – 130 kV) with a grid is now favoured, as this improves detail visibility in the higher attenuation mediastinum and produces better image quality over the whole image. When tube potentials of 100 kVp or above are employed for chest radiography, a high scatter fraction is produced and a high selectivity grid (12:1 to 18:1) should be used. An alternative method for reducing the level of scattered radiation in chest radiography is to use an air-gap of 200 mm or 300 mm between the patient and the radiographic cassette [35]. Scatter spreads out in all directions from the patient, whereas the primary beam spreads out from the X-ray tube focus. Thus when a gap exists between the patient and the film, a smaller proportion of the scattered radiation reaches the cassette. One effect of the air gap is to magnify the image, because the magnification is related to the ratio of the distances of the film and the patient from the X-ray tube. As a result, a larger focus to film distance of 3 m to 4 m is required when an air-gap is used to reduce the magnification to fit the whole image on the film. The output from the X-ray tube should also be increased to compensate for the greater distance. An air gap is not as effective as a grid in removing scatter and a slightly lower tube potential is required to achieve the same contrast level as a grid.

For paediatric examinations that have lower scatter levels, a lower grid ratio together with a lower tube potential that produce a better contrast level may provide a more satisfactory result. However, Aichinger et al [36] report that a grid with a high grid ratio (e.g., 15:1), if properly designed, can be better suited to paediatric radiography than a grid with a low ratio (e.g. 8:1), as recommended by the CEC [8], in which the thickness of the lead strips may be greater. Performance depends crucially on grid design.

### Beam collimation and X-ray projection

Collimation of the X-ray beam is an important factor in optimisation. Good collimation will both minimise the dose to the patient and improve image quality, because the amount of scattered radiation will increase if a larger volume of tissue is irradiated. Collimation is particularly important in paediatric radiography since the patient’s organs are closer together and larger fields are more likely to include additional radiosensitive organs. Collimation in most cases depends on the technique of the radiographer, but regular quality assurance by checking that the X-ray beam and the field from the light beam diaphragm are accurately aligned is important, particularly for mobile equipment.

**Figure 10:**
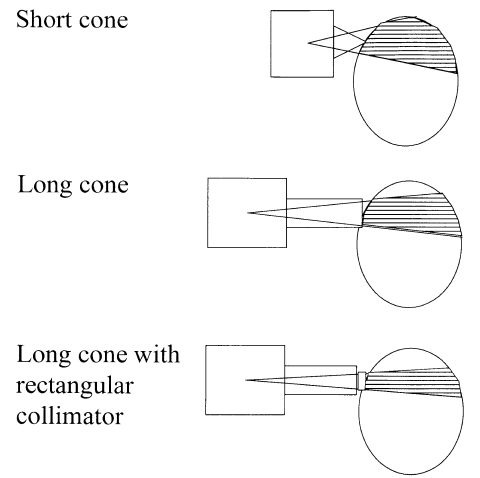
Arrangements for taking dental intra-oral radiographs with a short cone, a long cone and a long cone with a rectangular aperture, comparing the volume of tissue irradiated, which is shaded.

Beam collimation in dental radiography is achieved through use of a fixed cone and the traditional aperture size is 60 mm diameter [30, 37]. In older units which used a focus to film distance of 100 mm, a substantial proportion of the face was exposed. Optimisation has involved two stages, an increase in the focus to skin distance to 200 mm, and incorporation of a smaller rectangular aperture similar in size to the film. Both these have contributed to a reduction in the volume of tissue irradiated (Figure 10). However, the use of a smaller beam size means that alignment of the film is crucial. Therefore film holders placed in the mouth, with which the X-ray tube collimator can be aligned, should be used. Optimisation in dental radiography through the use of 65-70 kVp instead of 50 kVp, use of faster E speed film, and more accurate beam collimation can reduce the effective dose for a dental radiograph by a factor of ten.

**Figure 11:**
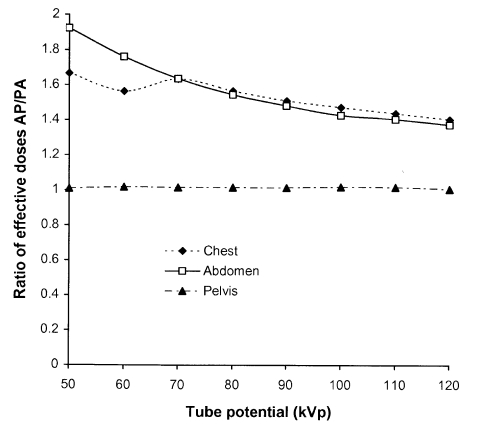
Ratios of effective doses for AP and PA projections for a reference patient as a function of tube potential for a selection of common radiographs [29].

Another aspect that influences the effective dose, is the projection chosen for a radiograph. The organs and tissues lying closer to the surface on which radiation is incident will receive higher radiation doses. If organs that are more sensitive to radiation are further from the surface on which the X-rays are incident, the X-ray beam will be attenuated by overlying tissues, and the doses to the organs will be lower. Therefore for some examinations the projection taken can influence doses to particular organs and the effective dose. Chest examinations will normally be taken using a postero-anterior (PA) projection, to minimise the dose to the breast tissue and oesophagus. Many of the abdominal organs are closer to the anterior surface, so a PA radiograph of the abdomen is also likely to have a lower effective dose. Effective doses for the antero-posterior (AP) view can be 50% higher for chest and abdomen radiographs, and even higher for low tube potentials, as shown by ratios of AP/PA effective doses for a few examinations plotted against tube potential (Figure 11). The discontinuity in the curve for chest examinations is a result of the method used for calculation of effective dose [14] and does not have any practical significance. Doses for AP and PA projections of the pelvis are similar, as the sensitive organs are located more centrally within the lower abdomen. Right and left lateral views of the pelvic area with similar exposure factors may also give different effective doses, since the exposure of the descending saecum will increase the dose to the colon for the right lateral view.

The risks from exposure to an embryo or foetus are greater than those to children or adults [14], so decisions involving investigations of pregnant women should be made carefully. The examination should only be performed if the risk of not making a diagnosis at that stage is greater than that of irradiating the foetus. Where the examination can be delayed without undue risk to the patient, this may be the better option, or if an acceptable technique using non-ionising radiation is available, this may be employed. If it is necessary to carry out a radiograph of the abdomen for a woman who is pregnant, the PA projection would reduce the dose to the foetus as much as possible.

### Film processing

The final stage in the production of a radiograph is processing the film. If processing conditions are not optimal, the film will require a higher radiation dose in order to provide an acceptable film density. Chemicals should be changed regularly, and the processing conditions, such as temperature and development time should be carefully optimised. A system of quality control that involves checking temperatures of processing chemicals and carrying out sensitometry, involving development of a test strip of film exposed to a range of light levels ensures optimal performance. These checks should be carried out daily to monitor performance in terms of film density, contrast and background fog level. The performance levels of processors that have a relatively low workload need to be monitored carefully. Since film processing affects the film density, it influences the speed index. Thus the measurements of the characteristic curve for a film, discussed earlier, will also reveal problems with processing.

**Figure 12:**
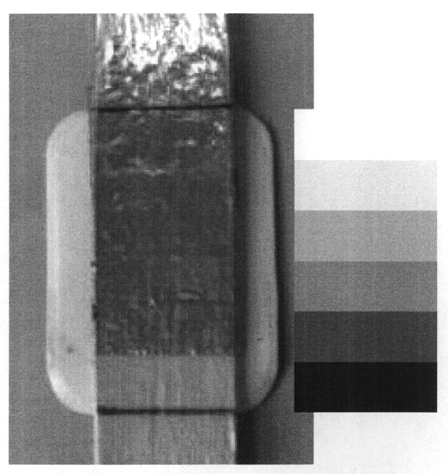
Simple dental step wedge test object with five different attenuation steps created using a dental spatula and different thicknesses of lead foil, taken from used dental film. A reproduction of the type of image that might be produced is also shown. The phantom is placed on top of the dental film at the end of the cone and an exposure taken with a standard setting. Subsequent exposures should be repeated under the same conditions, with the film placed on the same surface, as different scatter conditions may affect the image.

Processing can be a particular issue with dental radiography. A simple step wedge can be constructed using a spatula of low attenuation material with layers of lead foil taken from dental film. The step wedge with 0, 1, 2, 3, 4, and 5 thicknesses of lead foil can be placed on top of an intra-oral dental film and an exposure made with standard settings under standard conditions (Figure 12). If a film is taken with optimised processing, it can be considered the reference standard. Checks can then be made by comparing future results with the reference standard to identify any deterioration.

## DISCUSSION AND CONCLUSIONS 

The formation of images of the body involves interplay between many different factors. To achieve the correct balance between patient dose and image quality it is necessary to understand the way in which the images are formed, and to know the factors that influence the image quality and the radiation dose received by the patient, so that the appropriate options can be selected. The most important choice in radiography is the speed of the screen / film combination used. Rare earth systems with speed indices of about 400 are recommended for general radiography (Table 5) [8] and 600 may be appropriate for certain lumbar spine projections [22, 23]. Tests on the system speed should be carried out from time to time to check the performance, and if it is suspected that this may be a factor contributing to higher patient doses. Consistent exposures can be achieved using an AEC device. The AEC should be set up whenever a new type of screen / film system is introduced into a department, as sensitivities of different phosphors vary with tube potential in different ways. A simple shield device which allows each part of the film to be used for a single exposure enables film densities with different tube potentials and phantom thicknesses to be compared using a single film when setting up an AEC.

**Table 5:** Examples of good radiographic technique [8]

**Radiograph**	**Tube potential (kV)**	**Exposure time (ms)**	**Focal spot size (mm)**	**Speed index**
Skull AP/PA & LAT	70-85	<100	0.6	400
Chest PA	125	<20	≤1.3	400
Chest LAT	125	<40	≤1.3	400
Lumbar spine AP/PA	75-90	<400	≤1.3	400
Lumbar spine LAT	80-95	<1000	≤1.3	400
Lumbar spine LSJ	80-100	<1000	≤1.3	800
Pelvis AP	75-90	<400	≤1.3	400

Education in techniques for reduction of patient dose, coupled with periodic review of doses to feed back data to individual departments, provides the best way of achieving optimisation [6, 23]. Surveys of patient dose in terms of ESD or DAP and comparisons with DRLs should be carried out every few years [4]. When working in isolation, it is difficult to judge whether the dose to the patient for a particular examination is higher than it ought to be. The establishment of DRLs is a crucial step in optimisation as it enables hospitals to compare doses with established values to represent good practice. But if patient doses are found to be high, it is important that reasons are thoroughly investigated and shortfalls in equipment or technique addressed. There are many factors that can be involved, but the pattern of higher doses can give a clue to the possible reason. If doses for all examinations within a department are high, then the most likely cause is the screen / film combination used, or associated factors such as the processing. If certain X-ray units within a department have higher doses, this could be due to factors such as grid characteristics, incorrect alignment of the grid with the X-ray beam, or a relatively high table attenuation. If only certain examinations have higher doses, this may involve factors in technique, such as the choice of tube potential. In many cases if doses are above the DRL, there may be several factors involved.

The tube potential selected should be appropriate for the degree of contrast required and the thickness of the part of the body being imaged. The importance of grid characteristics and the interplay with exposure factors should not be forgotten. A higher tube potential gives rise to more scatter and requires use of a higher grid ratio. However, using a lower tube potential that produces less scatter with a lower grid ratio may result in better overall contrast for some examinations. Decisions about grid characteristics and whether a grid should be used in the first place should be based on the range of examinations performed. The use of low attenuation materials in couches and grids is particularly important for paediatric examinations, since the lower tube potential X-ray beams employed are more highly attenuated and the tissues of paediatric patients are more sensitive to radiation damage. Incorporation of an additional 0.1 mm or 0.2 mm of copper can give a significant reduction in ESD and should be considered, particularly for units used for paediatric and other examinations.

Dose reduction without regard for image quality could produce images that are inadequate for diagnosis and must be avoided. Alternative options for optimisation can be investigated using anthropomorphic phantoms or other phantoms designed to simulate imaging of detail within water, Perspex or other attenuating material designed to mimic clinical imaging situations. If any change in technique is introduced to optimise performance, an evaluation of a selection of clinical radiographs should be carried out, using criteria such as those in Table 4 to ensure that the image quality obtained is appropriate for the clinical task.

Despite all the effort to optimise radiography in recent years, the doses for similar examinations in different hospitals still vary substantially. However, the dose levels have gradually fallen as demonstrated by the gradual decline in the third quartile of survey data for the UK (Table 3) [10]. This has been achieved by carrying out optimisation through the performance of regular equipment quality assurance [24, 37] and periodic patient dose surveys to ensure that lower dose levels are maintained.
